# Inclusion, reporting and analysis of demographic variables in chronobiology and sleep research

**DOI:** 10.3389/fnins.2024.1421026

**Published:** 2024-09-16

**Authors:** Selma Tir, Rhiannon White, Manuel Spitschan

**Affiliations:** ^1^Department of Experimental Psychology, University of Oxford, Oxford, United Kingdom; ^2^Sleep and Circadian Neuroscience Institute, Nuffield Department of Clinical Neurosciences, University of Oxford, Oxford, United Kingdom; ^3^Warwick Medical School, University of Warwick, Coventry, United Kingdom; ^4^Department of Health and Sport Sciences, TUM School of Medicine and Health, Technical University of Munich, Munich, Germany; ^5^Translational Sensory and Circadian Neuroscience, Max Planck Institute for Biological Cybernetics, Tübingen, Germany; ^6^TUM Institute of Advanced Study (TUM-IAS), Technical University of Munich, Garching, Germany

**Keywords:** sleep, circadian rhythms, chronobiology, diversity, inclusion, reporting, demographics

## Abstract

Many aspects of sleep and circadian physiology are sensitive to participant-level characteristics. While recent research robustly highlights the importance of considering participant-level demographic information, the extent to which this information is consistently collected, and reported in the literature, remains unclear. This article investigates study sample characteristics within the published sleep and chronobiology research over the past 40 years. 6,777 articles were identified and a random sample of 20% was included. The reporting of sample size, age, sex, gender, ethnicity, level of education, socio-economic status, and profession of the study population was scored, and any reported aggregate summary statistics for these variables were recorded. We observed a significant upward trend in the reporting and analysis of demographic variables in sleep and chronobiology research. However, we found that while > 90% of studies reported age or sex, all other variables were reported in < 25% of cases. Reporting quality was highly variable, indicating an opportunity to standardize reporting guidelines for participant-level characteristics to facilitate Meta analyses.

## Introduction

Sleep and circadian rhythms are essential physiological and behavioral processes that can vary significantly among individuals (Horne and Östberg, 1977; Kerkhof, 1985; Tankova et al., 1994; Baehr et al., 2000; Van Dongen et al., 2005; Burgess and Fogg, 2008; Santhi et al., 2012). These variations manifest in sleep patterns, including the amount, timing, and quality of sleep, as well as in circadian rhythms, such as chronotype and circadian period. Some of these differences have been systematically linked to demographic variables, most notably age (Desforges et al., 1990; Bliwise, 1993; Benloucif et al., 2006; Espiritu, 2008; Duffy et al., 2015; Mander et al., 2017; Li et al., 2018), sex (Redline et al., 2004; Cain et al., 2010; Mong et al., 2011; Santhi et al., 2016; Anderson and FitzGerald, 2020), and ethnicity (Eastman et al., 2012, 2016; Goldstein et al., 2020; Ahn et al., 2021), demonstrating the need to consider participant-level characteristics in sleep and circadian studies. For instance, women tend to have a shorter circadian period (Duffy et al., 2011), are more likely to experience sleep disturbances and insomnia (Gordon et al., 2022), yet are less prone to sleep deterioration with aging compared to men (Redline et al., 2004). Moreover, considering sex-related factors, such as menstrual cycles, pregnancy, and menopause, can guide the development of sex-specific interventions, enhancing the diagnosis and treatment of sleep disorders in both sexes (Mallampalli and Carter, 2014). Ethnic disparities in sleep and circadian rhythms have also been documented. Certain racial and ethnic groups experience higher rates of sleep problems and circadian rhythm disruptions, such as African Americans and Hispanics/Latinos, compared to non-Hispanic Whites. These disparities are reflected in differences in circadian period, chronotype, phase shifting responses and sleep duration (Chellappa, 2021). Factors contributing to these disparities may include genetic predispositions and environmental exposures (Taheri and Mignot, 2002; Raizen et al., 2006). Understanding these multifaceted influences is thus critical for developing effective interventions.

Inequities in sleep health can also be linked to demographic variables such as education level, profession and socio-economic status (SES) (Raizen et al., 2006; Williams et al., 2015; Jean-Louis and Grandner, 2016; Laposky et al., 2016; Jackson and Johnson, 2020; Jackson et al., 2020a,b). These factors can influence an individual’s access to resources and better living conditions, which can, in turn, affect their sleep and circadian rhythms (Jehan et al., 2018). For example, it is suggested that individuals with lower SES are more likely to experience sleep disturbances and circadian rhythm disruptions compared to those with higher SES (Anders et al., 2014). This can be attributed to factors such as increased stress, demanding work schedules, and limited access to healthcare. Individuals with lower SES may also be more likely to reside in neighborhoods with higher levels of noise pollution and artificial light exposure, further compromising sleep quality (Casey et al., 2017). Similarly, different professions or employment statuses contribute to varying work schedules, demands, and stress levels. Individuals working night shifts, irregular schedules, or long hours often suffer from circadian misalignment and sleep difficulties, leading to adverse health outcomes (Wu et al., 2022). Additionally, geographical location may play a significant role in sleep and circadian rhythms research, as it influences environmental factors such as natural light exposure, temperature, altitude, noise levels, and air pollution (Okamoto-Mizuno and Mizuno, 2012; Halperin, 2014; Gupta et al., 2018; Blume et al., 2019; Liu et al., 2020). In hotter climates, the cultural practice of taking a midday nap or *siesta* can affect overall sleep patterns and alter the body’s internal clock (Monk et al., 2001; Lopez-Minguez et al., 2017). Some individual aspects of sleep and circadian physiology may thus be linked to genetic predispositions or influenced by cultural, environmental and societal factors (Park et al., 2023). Studies recruiting participants may be key to offer personalized solutions and treatments for sleep and circadian disruption. As compromised sleep has many knock-on effects, including negative effects on cardiovascular, metabolic, neurobehavioral and cognitive function, it is imperative to understand how demographic variables influence sleep and circadian rhythms.

It is suggested that research practices have historically excluded diverse populations at all stages of the research cycle, including recruitment, retention, data collection, analysis, and dissemination of findings (Taffe and Gilpin, 2021). This exclusion is particularly evident in the domain of sex. A recent study reviewing the reporting and analysis of sex in biological sciences research found that while the inclusion of sex as a variable has significantly increased over the past decade (Beery and Zucker, 2011; Woitowich et al., 2020), sex-based analysis has not improved correspondingly, despite recent policies and funding mandates promoting such practices (Institute of Medicine (US) Board on Population Health and Public Health Practice, 2012; Clayton and Collins, 2014). The term “gender data gap” has emerged to describe the historical exclusion of women from biomedical research (Heidari et al., 2016), highlighting a systemic bias in data collection and analysis that has led to a significant lack of understanding about sex-specific effects of diseases, treatments, and medical devices (Day et al., 2016). It is argued that this gap extends beyond healthcare, permeating numerous aspects of society where the lack of sex-disaggregated data has led to designs and policies that inadvertently disadvantage women. To address these gaps in biomedical research, several guidelines have been proposed, such as involving diverse communities in the design and implementation of research studies, employing culturally sensitive recruitment and retention strategies, and reporting demographic data in a transparent and standardized manner (Geller et al., 2018; Criado-Perez, 2019; National Institutes of Health, 2021). For instance, in 2016, the United States National Institutes of Health (NIH) issued a notice requiring grant holders to incorporate sex as a factor in the design, analysis and reporting of vertebrate and human studies, or to provide substantial justification for studying a single sex (Schiebinger, 2014). Similar disparities are prevalent in the biomedical and clinical research fields, where minorities are often understudied despite existing health inequities (Oh et al., 2015; Flores et al., 2021). A recent review of contemporary dementia research reported a lack of demographic, racial, and geographic diversity (Mooldijk et al., 2021). Additionally, an analysis of clinical trial populations found that 75% of participants were White for 53 drugs approved by the US Food and Drug Administration (FDA) in 2020 (U.S. Food and Drug Administration, 2020). These findings collectively underscore the critical need for representative study populations in order to develop effective, equitable, and tailored interventions to promote healthy sleep and circadian rhythms across all demographics.

While participant-level demographic characteristics significantly impact outcomes, the extent to which this information is consistently collected, and reported in the literature, remains unclear (Artiga et al., 2020). To address this gap, we conducted a comprehensive analysis of whether participant-level demographic characteristics (age, sex, gender, ethnicity, level of education, socio-economic status, and profession of the study population) are reported and analyzed in chronobiology and sleep research. Our study examined 1355 randomly sampled publications from the eight top-ranked chronobiology and sleep research journals, as determined by Journal Impact Factor, over the past forty years. We systematically extracted the study sample characteristics and evaluated the inclusion, reporting, and analysis of demographic variables, thereby assessing the representativeness of findings within the field. This study aims to provide insights into the historical trends and current practices in demographic reporting, highlighting areas for improvement to promote inclusivity and diversity in future research. By identifying gaps in demographic representation and reporting, our study contributes to the ongoing efforts to enhance the quality and applicability of sleep and circadian rhythm research across diverse populations.

## Methods

### Procedure

Journal articles published between 1979 and 2019 in the top eight sleep and chronobiology journals were considered. For practical reasons, a temporal resolution of 2 years was considered sufficient to determine any effects changing over time, and the number of screened articles was reduced by only analyzing those published in odd years. The list of possible target journals was based on a previously established list of journals implementing a hybrid strategy by consulting the Web of Science Master Journal List, domain-relevant expertise in sleep and chronobiology and consulting with a senior researcher with > 25 years of experience in the field (Spitschan et al., 2020). From this previously derived list, we selected eight journals based on their five-year Impact Factor, and included *Journal of Pineal Research* (ISSN: 0742-3098 / 1600-079X; 2018 5-year IF: 12.197), *Sleep* (0161-8105 / 1550-9109; 5.588), *Journal of Sleep Research* (0962-1105 / 1365-2869; 3.951), *Sleep Medicine* (1389-9457 / 1878-5506; 3.934), *Journal of Clinical Sleep Medicine* (1550-9389 / 1550-9397; 3.855), *Journal of Biological Rhythms* (0748-7304 / 1552-4531; 3.349), Behavioral Sleep Medicine (1540-2002 / 1540-2010; 3.162), and *Chronobiology International* (0742-0528 / 1525-6073; 2.998). While *Sleep Medicine Reviews* also features in the list of journals, we did not include it as it primarily publishes reviews.

### Article inclusion

6,777 articles were identified through a MEDLINE search and filtering by journal and odd years. A random sample of 20% was initially selected for screening. Inclusion requirements included conducting original research in the English language, reporting human data, and recruiting volunteers. As such, animal studies, bibliographies, case reports, comments, conference proceedings, editorials, guidelines, letters, retracted publications, reviews, errata and corrigenda were excluded.

### Review and article extraction

All included articles were reviewed for eligibility and coded by RW. The reporting of sample size, age, sex, gender, race/ethnicity, level of education, socio-economic status, and profession of the study population was scored binarily (0 = not reported, 1 = reported), and any reported aggregate summary statistics for these variables were recorded (e.g., mean, median, etc.). Sample size referred to the total number of participants for a given study. If an article reported multiple studies, then it was analyzed for each of its individual studies. Age was analyzed when recorded in days, weeks or years. Sex referred to biological sex, while gender referred to the social construction of sex. When sex and gender were used interchangeably and didn’t refer to personal identification, it was scored as biological sex. Since the language and system describing ethnicity, level of education, socio-economic status and profession differ between countries and individuals, all reporting was taken into account as long as there was a clear indication of what the variable represented. For example, socio-economic status included categories of income, seniority within a company or type of labor, and “occupation” and “employment status” were recorded as profession. Additionally, the non-demographic variables, funding source, geographical location and clinical focus of the article, were examined, as well as whether data were analyzed by including any of the demographic variables as covariates. Data were coded in an Excel Spreadsheet and analyzed in R Studio (version 4.2.2).

### Pre-registration

We pre-registered our protocol using the PRISMA-P template (Moher et al., 2015; Shamseer et al., 2015) on the Open Science Framework.^1^ For thorough details on the screening and analysis methods, see the protocol.

### Materials, data and code availability

All data underlying this manuscript are available on a public GitHub repository.^2^ The article was written in R (R Core Team, 2020) using RMarkdown and papaja (Aust and Barth, 2020), employing a series of additional R packages (Wickham, 2007, 2016, 2019; 2021; Xie, 2015; Auguie, 2017; Wei and Simko, 2017; Wickham and Bryan, 2019; Kassambara, 2020; Wilke, 2020; Wickham et al., 2021; Barth, 2022) and is fully reproducible.

## Results

### Number of analyzed articles

**FIGURE 1:**
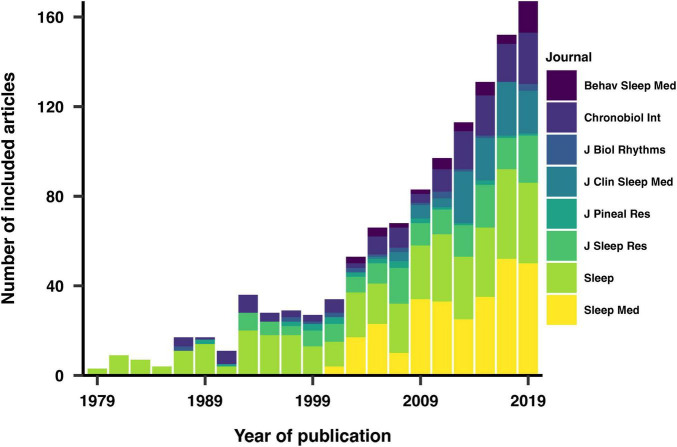
Distribution of included and analyzed articles by year and journal of publication. The increasing representation of recent articles reflects a rise in scientific output over time.

From an initial pool of 1355 identified and pre-screened articles, we included and extracted data from 1152 (85%), adhering to our inclusion criteria. The distribution of publication years was non-uniform, with a higher proportion of articles from more recent years being included (Figure 1). The representation of journals in the final list was also non-uniform, as not all journals have been consistently available from the start of our data collection in 1979.

We also investigated the reasons for exclusion among the 203 articles that were not included in our analysis. The primary reason for exclusion across the years was the lack of original research content, followed by the absence of participant recruitment and the reporting of non-human data. The exclusion criteria varied slightly over different years, reflecting changes in publication practices and research focus over time.

### Funding

**FIGURE 2:**
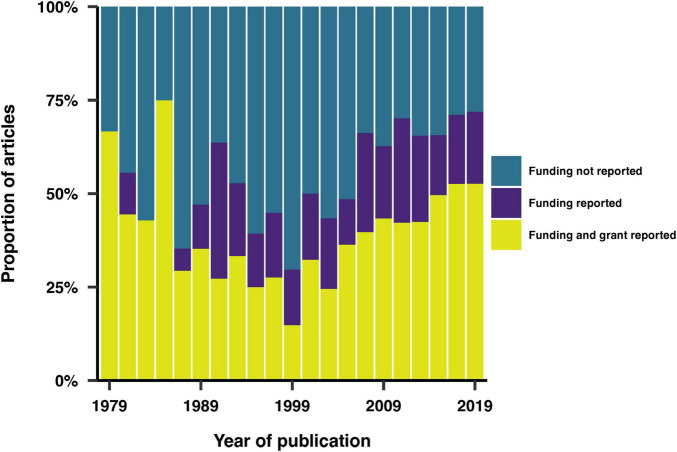
Trends in the reporting of funding sources and grant codes over time.

Our examination of the reporting of funding sources in the included articles revealed that 62% of the studies disclosed their funding sources, with 69% of these also providing specific funding numbers (Figure 2). The United States National Institutes of Health (NIH) was the most reported funding agency, representing 19% of the reported sources. Notably, 92% of NIH-funded studies also reported their specific funding numbers. The Australian National Health and Medical Research Council (NHMRC) and the Canadian Institutes of Health Research (CIHR) were the second most frequently mentioned funding agencies.

### Geographical location

**FIGURE 3:**
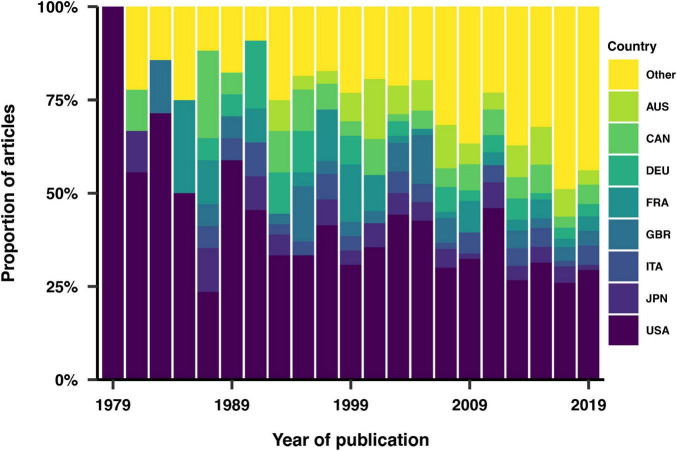
Geographical distribution of the studies. The eight most represented countries across the dataset are individually highlighted. AUS, Australia; CAN, Canada; DEU, Germany; FRA, France; GBR, United Kingdom of Great Britain and Northern Ireland; ITA, Italy; JPN, Japan; USA, United States.

Of the analyzed articles, 93% were conducted within a single country. Explicit reporting of the study’s geographical location was found in 57% of the articles. For the remaining articles, the country of the study was inferred, primarily based on the affiliation of the first author, as this typically indicates the institution where the research was conducted. We also assumed that if the study population differed from the institution’s geographic location, it would be explicitly stated in the article. 53 countries were represented overall. Figure 3 shows the distribution of study locations over time, highlighting the eight most represented countries. The United States consistently emerged as the most represented country across all years. In contrast, only three studies were primarily conducted in Africa, specifically in Nigeria, Senegal and Tunisia.

### Sample size

**FIGURE 4:**
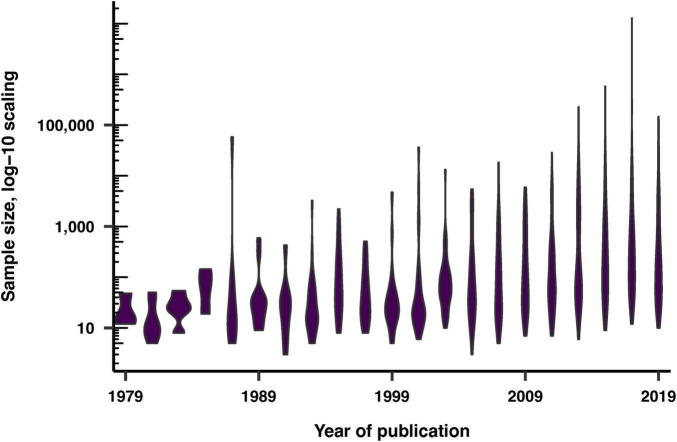
Sample sizes of the recruited volunteers over publication years, displayed on a log-10 scale.

We examined the reporting of sample sizes in the studies, documented these figures, and investigated their distribution as a function of the publication year of the articles. Sample sizes were reported in 92% of the studies, with a wider distribution of sample sizes in more recent articles (Figure 4).

### Age

**FIGURE 5:**
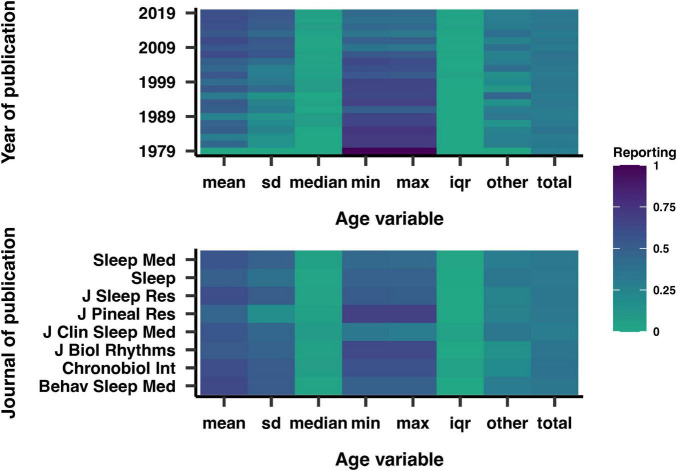
Reporting of various age-related variables by year (top) and journal (bottom) of publication. Darker shades indicate a higher correlation. SD, standard deviation of the mean; IQR, interquartile range.

93% of articles reported a variable describing age, such as the mean, standard deviation, median, minimum, maximum, and interquartile range. Among these, the median and interquartile range were the least reported variables. Figure 5 shows the trends in the reporting of these variables over the years. In 1979, the minimum and maximum ages were the most commonly employed variables, but their usage decreased over time relative to other variables. Conversely, the reporting of the standard deviation of the mean age increased throughout the years. Specific trends were also observed across different journals, such as the frequent use of the minimum and maximum age variables in *Journal of Pineal Research*, and the lack of in *Journal of Clinical Sleep Medicine*.

**FIGURE 6:**
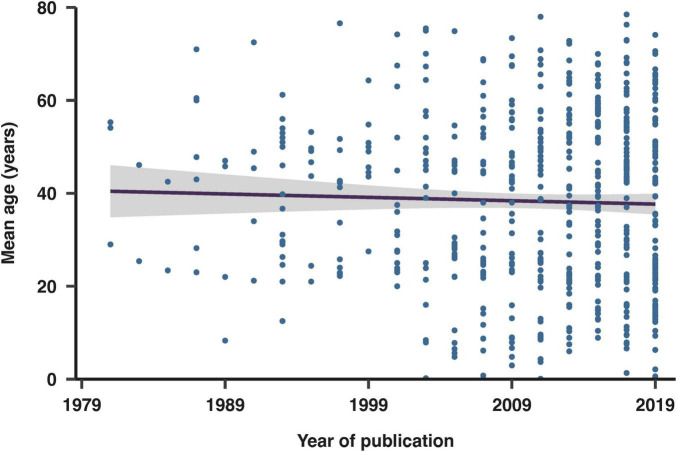
Evolution of mean age in included studies over publication years. The fit shown is a linear regression with 95% confidence intervals.

Overall, the average mean age of the study populations was 39 years old. We investigated how the mean age varied across studies over time (Figure 6), and observed that the mean age distribution became more diverse in recent years. While the range of ages significantly expanded over the years, the mean age remained relatively constant, centered around the 40 year old population.

### Sex and Gender

**FIGURE 7:**
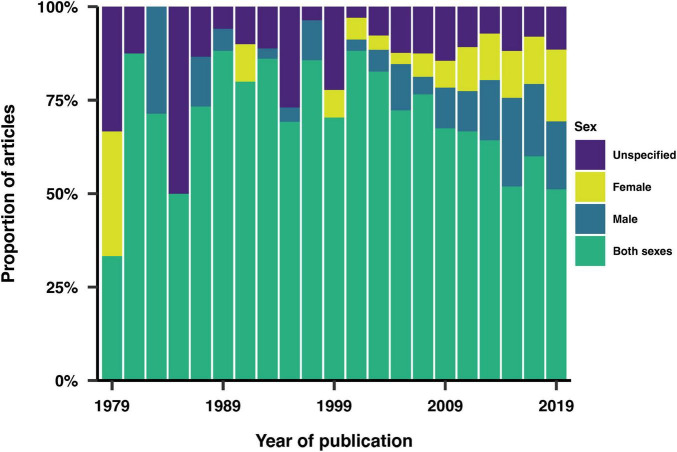
Sex inclusion over time. Proportion of studies that recruited male subjects, female subjects, both sexes, or did not specify the sex of the participants.

Sex was reported in 89% of the studies. Figure 7 illustrates the proportion of studies that recruited male subjects, female subjects, both sexes, or did not specify the sex of the participants. 13% of the studies that reported sex recruited only male participants, while 10% recruited only female participants. Among the studies focusing on a single sex, 1% of those involving males and 2% of those involving females examined sex-dependent features. These studies focused on conditions that exclusively impact one sex, such as prostate cancer, erectile dysfunction, and menstrual disorders. Additionally, 4% of studies reported age data disaggregated by sex. Gender was reported in only one article, which categorized participants as male, female, or transgender.

### Ethnicity, education, profession and socio-economic status

**FIGURE 8:**
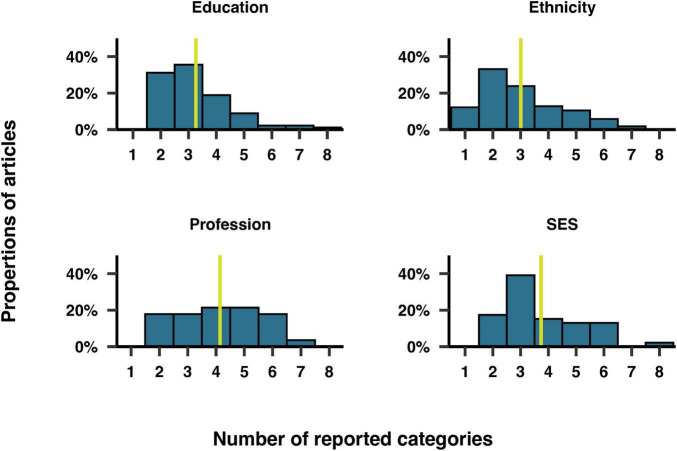
Trends in the reporting of education, ethnicity, profession and socio-economic status by year of publication.

We examined the reporting of additional demographic variables, including race/ethnicity, education, profession and socio-economic status (SES). We found that these demographic variables were reported in 15% for ethnicity, 12% of studies for education, 4% for socio-economic status, and 2% for profession. Figure 8 shows the distribution of this reporting across the years. Qualitatively, there is a clear increase in the reporting of additional demographic variables over time, with ethnicity being the most reported demographic variable in more recent years.

**FIGURE 9:**
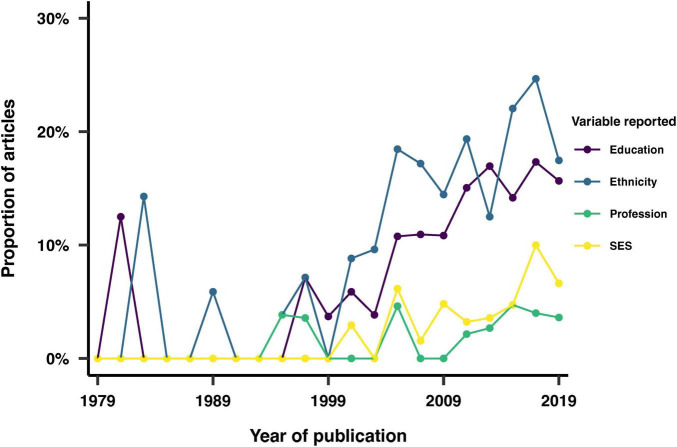
Distribution of the number of categories reported for education, ethnicity, profession and socio-economic status (SES). The yellow vertical line corresponds to the median.

Furthermore, we examined the number of categories included for each of these demographic variables. Figure 9 illustrates the number of categories reported for each variable among the articles that included them. On overage, the coding scheme for each variable comprised three to four categories. Education was reported as a range of years of study, degree level, or arbitrary categories such as “low, medium, high.” The mean number of years of education was reported in 34% of articles, averaging 14 years of study overall. Participants could choose multiple categories for ethnicity in 6% of the studies reporting this variable. The most common categories for ethnicity were “White/Caucasian,” “Black/African American” and “other,” mentioned in 89%, 60%, and 53% of studies, respectively. The most common category for profession was “unemployed,” mentioned in 39% of studies, along with a variety of specific jobs, such as attorney, farmer and astronaut. In 57% of studies reporting SES, the categories of SES were based on income ranges. The remaining articles reported SES using arbitrary categories such as “high, medium, low.”

### Study focus

We considered whether each article reported on a specific, pre-defined group of people. Our analysis revealed that 3% of the articles focused on a sex-dependent feature, while 50% investigated a clinical feature, such as sleep apnea. Additionally, 1% of the studies focused on twins, 1% on pregnant women, 2% on shift workers, and 4% on university students.

### Analysis disaggregation

**FIGURE 10:**
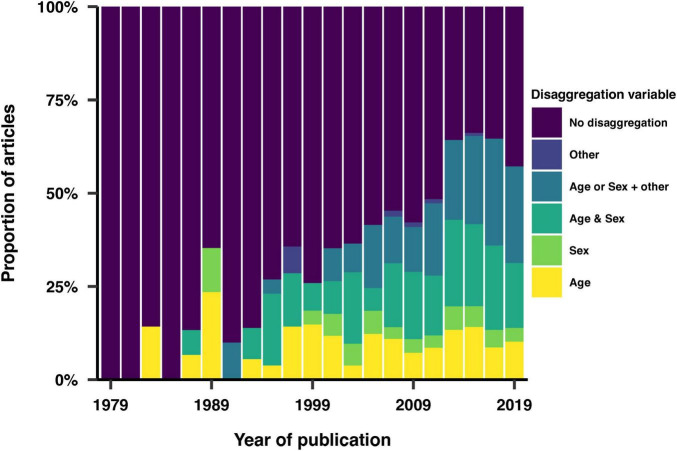
Use of study population characteristics as variables in the analysis.

We investigated the extent to which articles reported subgroup analyses of the data based on one or more of the reported demographic variables. We found a significant increase in the frequency at which subgroup analyses of the study samples were performed over time (Figure 10). The most common subgroup analyses involved disaggregating by sex, age, or both.

## Discussion

### Summary of main findings

This review analyzed the inclusion and reporting of 1,152 articles in chronobiology and sleep research, sampled from eight journals. We observed a higher inclusion rate for more recent articles, although the representation of journals was uneven. The non-uniformity of this representation can be attributed to the increasing volume of research in the field of sleep and circadian rhythms, the availability of journals over time, and variations in their annual publication volumes. The studies predominantly originated from North America and central Europe, with the United States being the most represented country. In contrast, only 0.3% of the screened articles came from Africa, highlighting a significant gap in geographical inclusion. This geographic skew raises concerns about the applicability of findings to diverse populations globally and underscores the need for more inclusive research efforts that encompass a broader range of geographical contexts. Over time, we observed an increase in sample size ranges and a broader age distribution in study populations. This pattern likely reflects a trend towards including larger and more variable sample sizes, as well as a broader consideration of age diversity in study samples.

Across the last forty years of sleep and chronobiology research, almost 90% of all screened articles reported sex, while at least half of the studies reported data from both sexes. This high reporting rate highlights the growing recognition of the importance of sex as a biological variable. Additionally, differentiating between sex and gender may allow for a more comprehensive understanding of the potential differences in health outcomes between males and females, and the impact of social and cultural factors related to gender identity. Sex refers to the biological differences between males and females, whereas gender encompasses the social and cultural roles, behaviors, and identities associated with being male or female. Incorporating both sex and gender considerations can help ensure that treatments are safe and effective for all individuals, regardless of their sex or gender identity. In this study, we found that only one article reported gender and categorized participants as male, female, or transgender. This finding may suggest a gap in the inclusion of gender identity and the transgender population in sleep and circadian rhythms research.

We observed an upward trend in the reporting of additional demographic variables (ethnicity, education, profession and SES), with ethnicity being the most frequently reported variable in recent years. Profession refers to an individual’s occupation or employment status, while SES refers to an individual’s or a family’s social and economic standing. Education level, profession and SES can influence access to resources, opportunities, and living conditions, which in turn affect sleep and circadian rhythms. Understanding the impact of these variables can help identify groups that may be at higher risk for sleep-related conditions, and inform interventions to improve sleep and circadian rhythms in these populations. It can also help reduce health disparities by addressing underlying cultural, educational, socioeconomic and work-related factors. The diverse array of reporting strategies, which included both objective and arbitrary measures, indicates an evolving and nuanced approach to capturing demographic data. Objective measures provide standardized and quantifiable data, which are essential for rigorous analysis and comparison across studies. On the other hand, subjective or arbitrary measures can offer deeper insights into personal and contextual factors that influence sleep and circadian rhythms, thereby enriching our understanding of these complex phenomena. Whether one strategy provides advantages over the other remains to be decided.

Disaggregating data by demographic factors allows researchers to identify patterns and associations that may not be apparent when analyzing the data as a whole. Our review shows that analysis disaggregation was increasingly used over the years and involved over half of the screened articles in the last decade. The most common subgroup analyses involved disaggregating data by sex, age, or both. This trend underscores the field’s movement towards more detailed and precise analyses, which can reveal critical insights into how different demographic groups experience sleep and circadian disruptions.

### Taking an inventory of represented study samples

The applicability of scientific findings to wide and diverse populations heavily relies on the representativeness of study samples across various demographic categories. The question to what extent the composition of a given study sample can make the applicability of findings difficult or impossible has received attention in the field of psychology, where many articles published in prominent journals reflected participants from WEIRD (Western, Educated, Industrialized, Rich, and Democratic) contexts (Henrich et al., 2010; Muthukrishna et al., 2020). In other fields, analyses similar to the one in the present review have been published (Sifers, 2002; O’Bryant et al., 2004; Jones et al., 2020; Flores et al., 2021), but to our knowledge, this review represents a first look at the inclusion, reporting and analysis of participant demographics in chronobiology and sleep research.

### The need to consider individual differences

It is well-established that health, sleep, and circadian physiology exhibit substantial individual differences, rendering a one-size-fits-all approach ineffective for promoting healthy sleep and circadian rhythms (Regitz-Zagrosek, 2012; Mallampalli and Carter, 2014; Grandner, 2017; Billings et al., 2021). Demographic variables offer a crucial perspective for understanding these individual differences, and can importantly illuminate systemic disadvantages and inequities. In the clinical domain, the need to tailor therapy timing to a patient’s individual circadian rhythm has given rise to the emerging field of chronotherapy or chronotherapeutics (Adam, 2019; Dijk and Duffy, 2020; Greco and Sassone-Corsi, 2020; Hill et al., 2020). Given the inherent variability and complexity of human sleep and circadian rhythms, it becomes evident that extracting an unbiased set of data representing “normal” sleep is exceedingly difficult, if not impossible. The diversity in sleep patterns across different ages, sexes, ethnicities, and socio-economic backgrounds underscores the necessity of personalized approaches. Understanding interindividual variability should become a central research focus to comprehend circadian and sleep physiology within the context of human diversity.

Statistical associations between socio-demographic variables can arise, demonstrating the complex relationships between participant-level characteristics within populations. For instance, in a study on a specific occupational group, other socio-demographic variables might seem redundant to report if the occupation inherently requires a particular education level or is predominantly filled by one gender. However, assumptions about the uniformity of demographic variables within specific populations can obscure the accurate reporting and analysis of these variables, undermining the commitment to inclusivity and diversity. Depending on the statistical model, socio-demographic variables can also be included within interaction terms. However, while using socio-demographic variables as covariates might help adjust for potential confounding, it does not fully capture the complex interplay between variables. Therefore, accurate demographic reporting is crucial to reflect demographic variables that might drive or moderate outcomes variables. Additionally, researchers might opt not to include all demographic variables due to assumed correlations. We do not believe that this speculative approach is sufficient, unless two demographic variables fully overlap in all situations. Comprehensive demographic reporting ensures a nuanced understanding of the relationships between socio-demographic variables and study outcomes, enhancing the validity and applicability of research findings.

### Advancing inclusion and diversity of study populations

Efforts to address gaps in inclusion and diversity within sleep and circadian rhythms research have been initiated (Day et al., 2017; Tannenbaum et al., 2019). Funding agencies now often require or strongly encourage researchers to report demographic characteristics of study participants in grant applications and research publications. Similarly, many scientific journals mandate the reporting of study populations’ demographic characteristics, including age, sex, and race/ethnicity. Some journals have also implemented specific policies to promote inclusion and diversity in research, such as requiring authors to address any potential biases in their study design (Jack et al., 2023). Additionally, researchers are increasingly collaborating with community organizations to recruit study participants from underrepresented groups. These efforts are reflected in our paper’s findings, which highlight an upward trend in the reporting of demographic variables over time. The increased inclusion of variables such as ethnicity, education, profession, and SES in recent articles underscores the growing commitment within the field to enhance the representativeness of research findings.

### Limitations of the current review

We turn to possible limitations of this review and the included analyses and discuss how they might introduce bias in our findings. In response to concerns about potentially missing relevant sections of the literature, we acknowledge the inherent limitations of any literature review in terms of comprehensiveness. To provide an estimate of uncertainty, we considered several factors. First, our sampling methodology involved a random selection of articles from the top eight journals in the field, which helps mitigate selection bias but cannot entirely eliminate it. The chance of missing relevant articles is estimated to be low, given the large sample size and inclusion of leading journals. However, our focus on top-ranked journals may exclude research published in inter-disciplinary or lesser-known journals, potentially missing 5–10% of relevant studies. Although we considered randomly sampling a subset of chronobiology and sleep research articles using a general search (e.g. on search from “sleep OR chronobiology” on MEDLINE), we deemed this approach too permissive. Selecting a subset of candidate journals offered a reasonable trade-off, ensuring a focus on field-specific outlets while maintaining a manageable scope. Additionally, while our review covers publications over the past forty years, older studies may be underrepresented due to less rigorous archiving and digitization practices in earlier decades, with an estimated uncertainty of around 5%. Due to the non-uniform distribution of publication years among the included articles (Figure 1), variables derived from published papers and visualized or analyzed by year will have varying degrees of uncertainty, with earlier years exhibiting higher uncertainty due to fewer articles. However, this uneven representation is a reflection of the exponential growth of scientific output over time, rather than a flaw in our dataset (Bornmann and Mutz, 2015; Parolo et al., 2015; Powell et al., 2017). Lastly, our selected journals are predominantly based in North America and Europe, introducing a geographical and language bias that could account for 10–15% of potentially relevant studies not included.

We also consider the potential effect of publication bias and how it could manifest in our review. Studies with positive or significant results are more likely to be published, leading to an overrepresentation of such findings and skewing the understanding of the true variability and strength of associations. High-impact journals, which we selected, often favor novel and significant results, potentially underrepresenting replication or non-significant studies. Additionally, the dominance of studies from North America and Europe in our sample may reflect regional publication biases, where research practices and priorities differ from other parts of the world. Overall, we estimate that the uncertainty in our literature review might result in missing approximately 10% of relevant studies.

### Towards standardized reporting of demographic variables: From checklists to schemas?

There are existing guidelines and checklists, such as CONSORT (Schulz et al., 2010) or STROBE (von Elm et al., 2007), for standardizing the reporting of participant characteristics. Resources like the Equator Network^3^ offer extensive databases of health research reporting guidelines. Some biomedical journals (e.g. (Robinson et al., 2017) specify demographic reporting requirements in their author instructions. Additionally, organizations may recommend specific reporting items for particular study questions (Veitch and Knoop, 2020). Adhering to these guidelines and checklists can ensure the reporting of relevant and comprehensive participant information, thereby improving the reproducibility and transparency of research, facilitating data sharing and integration across different studies and datasets, and promoting more equitable and inclusive sleep and chronobiology research.

Yet, these guidelines and checklists primarily focus on *what* should be reported and not how it should be reported. There is, *a priori*, however, no reason not to develop and use standardized, machine-readable schemas for reporting participant characteristics. The FAIR principles advocate that data should be findable, accessible, interoperable, and reusable (Wilkinson et al., 2016). One way to achieve these criteria is through the use of data schemas that prescribe categories of data and common naming schemes for reporting participant characteristics. It is crucial, however, to understand that “what gets counted counts” (D’Ignazio and Klein, 2020), and to ensure that such data schemas are not exclusionary, e.g., by enforcing sex binaries (Hyde et al., 2019), and to critically assess whether specific demographic variables are truly important, following the principle of data minimization. Additionally, careful consideration is needed to ensure that disaggregation by demographic variables is not used in ways that could cause harm (D’Ignazio and Klein, 2020).

## Conclusion

This review provides a first look at the inclusion, reporting and analysis of demographic variables in the chronobiology and sleep research literature, evaluating over 1,000 articles across eight specialized journals. Our findings address the need to consider individual differences, as well as the dependence of sleep and circadian rhythms on demographic variables. We observed significant progress over time in the reporting of demographic variables such as ethnicity, education, profession, and socioeconomic status. However, variability in reporting methods indicate a need for standardization to improve data comparability and research reproducibility. Furthermore, we identify an opportunity to improve the reporting of participant-level characteristics through the adoption of formalized data schemas. Our review underscores the necessity for continued and enhanced efforts to diversify study populations in chronobiology and sleep research, ensuring that research findings are applicable to and beneficial for all segments of the population, thereby advancing the field in a more inclusive and equitable direction.

## Research agenda

Future research needs to: 1. Establish schemas for reporting demographic variables in a harmonized way across geographical and cultural contexts; 2. Identify gaps in the sleep and chronobiology literature with respect to understudied populations; 3. Understand the extent to which research practices allow for the inclusion of diverse populations in all stages of the research cycle, and how this can exacerbate health inequities.

## Practice points

1. Published studies on circadian and sleep physiology should be carefully examined.

2. Reporting of demographic variables should be done deliberately and systematically.

3. Inclusion and diversity of different populations across the field needs to be ensured.

## Footnotes

1
https://doi.org/10.17605/osf.io/cu3we

2
https://github.com/tscnlab/TirEtAl_FrontNeurosci_2024

3
https://www.equator-network.org/
